# Multiple Insecticide Resistance in *Anopheles gambiae* s.l. Populations from Burkina Faso, West Africa

**DOI:** 10.1371/journal.pone.0048412

**Published:** 2012-11-26

**Authors:** Moussa Namountougou, Frédéric Simard, Thierry Baldet, Abdoulaye Diabaté, Jean Bosco Ouédraogo, Thibaud Martin, Roch K. Dabiré

**Affiliations:** 1 Institut de Recherche en Sciences de la Santé/Centre Muraz, Bobo-Dioulasso, Burkina Faso, West Africa; 2 Maladies Infectieuses et Vecteurs: Ecologie, Génétique, Evolution et Contrôle, Institut de Recherche pour le Développement, Montpellier, France; 3 Centre de Recherche Entomologique de Cotonou, Cotonou, Bénin; 4 Centre de Coopération Internationale en Recherche Agronomique pour le Développement, Montpellier, France; University of Crete, Greece

## Abstract

Malaria control programs are being jeopardized by the spread of insecticide resistance in mosquito vector populations. The situation in Burkina Faso is emblematic with *Anopheles gambiae* populations showing high levels of resistance to most available compounds. Although the frequency of insecticide target-site mutations including *knockdown resistance (kdr)* and insensitive acetylcholinesterase (*Ace-1* ^R^) alleles has been regularly monitored in the area, it is not known whether detoxifying enzymes contribute to the diversity of resistance phenotypes observed in the field. Here, we propose an update on the phenotypic diversity of insecticide resistance in *An. gambiae* populations sampled from 10 sites in Burkina Faso in 2010. Susceptibility to deltamethrin, permethrin, DDT, bendiocarb and fenithrotion was assessed. Test specimens (N = 30 per locality) were identified to species and molecular form and their genotype at the *kdr* and *Ace-1* loci was determined. Detoxifying enzymes activities including non-specific esterases (NSEs), oxydases (cytochrome P450) and Glutathione S-Transferases (GSTs) were measured on single mosquitoes (N = 50) from each test locality and compared with the *An. gambiae* Kisumu susceptible reference strain. In all sites, mosquitoes demonstrated multiple resistance phenotypes, showing reduced mortality to several insecticidal compounds at the same time, although with considerable site-to-site variation. Both the *kdr* 1014L and *Ace-1* ^R^ 119S resistant alleles were detected in the M and the S forms of *An. gambiae*, and were found together in specimens of the S form. Variation in detoxifying enzyme activities was observed within and between vector populations. Elevated levels of NSEs and GSTs were widespread, suggesting multiple resistance mechanisms segregate within *An. gambiae* populations from this country. By documenting the extent and diversity of insecticide resistance phenotypes and the putative combination of their underlying mechanisms in *An. gambiae* mosquitoes, our work prompts for new alternative strategies to be urgently developed for the control of major malaria vectors in Burkina Faso.

## Introduction

Malaria remains one of the most critical public health challenges for Africa despite intense national and international efforts [1]. Indoor Residual Spraying (IRS) and Long Lasting Insecticide treated Nets (LLINs) are the primary tools for malaria vector control. Several studies have demonstrated the efficacy of both tools in curbing malaria incidence [2], [3]. However, emergence and spread of insecticide resistance in major mosquito vector species could jeopardize the success of malaria control programs [4]. Resistance to the four major classes of insecticides available for public health has indeed been reported in the primary African malaria vector, *Anopheles gambiae sensu lato* [5], [6]. Reports of insecticide resistance in mosquito vector populations in Burkina Faso appeared as early as the 1960s, when *An*. *funestus* and *An*. *gambiae* s.l. (thereafter, *An. gambiae*) populations that showed reduced mortality to dieldrin and DDT were described [7], [8]. Recent studies have confirmed that resistance to DDT is still prevailing at a high level in *An*. *gambiae* populations from Burkina Faso, where resistance to pyrethroids was also increasingly reported [9], [10], [11]. Today, pyrethroid insecticides are the most widely used compounds in public health because of their high effectiveness and strong excito-repellent effect on insects, as well as low mammalian toxicity [12], [13]. Unfortunately resistance to this insecticide class is now widespread in *An. gambiae* [9], [10], [14], [15]. Mutations in the gene encoding the voltage-gated sodium channel [16] have been shown to be an important mechanism providing mosquitoes with high levels of cross-resistance to pyrethroids and DDT which share the voltage-gated sodium channel as a target site. The *knock-down resistance (kdr)* mutation, changing a Leucine (TTA) to a Phenylalanine (TTT) at position 1014 of the voltage-gated sodium channel gene (i.e. L1014F mutation) was predominant in West and Central African *An. gambiae* populations [9], [17] whereas another substitution, changing the Leucine (TTA) to a Serine (TCA) at position 1014 (i.e. L1014S mutation) originated from Kenya [18], has now spread into Central Africa including Cameroon [19], [20], Equatorial Guinea [21] and Gabon [22]. More recently this mutation was detected in *An. arabiensis* in Benin, West Africa [23].

There is therefore an urgent need to investigate alternatives to the current reliance on pyrethroids for malaria vector control. In the short term, other insecticide classes with different modes of action such as organophosphates (OPs) and carbamates (CMs) could be used either alone or in combination with pyrethroids for IRS or for impregnating bednets [24]. However, resistance to both classes of insecticides has already been documented in natural *An. gambiae* s.l. populations. This resistance to OPs and CMs in *An. gambiae* is due to a single point mutation in the *Ace-1* gene encoding acetylcholinesterase, the target binding site of OPs and CMs, resulting in the substitution of a Glycine (GGC) into a Serine (AGC) at position 119 of the encoded protein (i.e. G119S mutation) [25], [26]. At first, reduced susceptibility to OPs and CMs was described in *Culex* populations from Côte-d'Ivoire [27]. More recently it was observed in *An. gambiae* populations in the centre and the north of Côte d'Ivoire [28], in Benin [29] and in Burkina Faso [30], [31].

Insecticide resistance may also occur by other physiological mechanisms such as metabolic detoxification through increased enzyme activities (monooxygenases, esterases, or glutathione S-transferases) [32], [33]. Increased glutathione S-transferases activity in mosquitoes typically confers resistance to the organochlorine insecticide DDT [34], [35], and can act as a secondary mechanisms for OPs resistance [36]. Esterases produce a broad spectrum of resistance in many *Culex* species, but in *Anopheles* esterase-based resistance is usually specific to the OP malathion [37], [38]. Pyrethroid resistance in *An. gambiae* in East and West Africa appears to be linked to increased monooxygenase titres, in the latter case combined with an altered target-site mechanism [29], [39]. A recent study carried out in Benin using biochemical assays incriminated detoxifying enzymes in conferring resistance to permethrin, DDT, dieldrin and carbosulfan in *An. gambiae* and *Culex quinquefasciatus* [29]. In Cameroon, work using biochemical assays showed metabolic detoxification is a major and widespread DDT and pyrethroid resistance mechanism in *An. gambiae* [40]. In Benin and Nigeria microarray analysis showed that two cytochrome P450s namely CYP6 P3 and CYPM2 were mainly incriminated in pyrethroid resistance in *An. gambiae* s.s. populations [41]. In Cameroon resistant *An. gambiae* s.s. populations overexpressed CYP6 P3 [42] whereas resistant *An. arabiensis* populations overexpressed CYP4G16 together with glutathione S-transferase GTS1 and a number of antioxidant genes [43].

The situation of multiple resistance in *An. gambiae* found in moist savannas of western Burkina Faso [44] with the concomitant presence of *kdr* L1014F and *Ace-1* G119S mutations is particularly alarming and constitutes a major threat to the successful outcome of current malaria control strategies. However, to date, no study has investigated the role of metabolic-based detoxifying mechanisms in contributing to insecticide resistance in *An. gambiae* populations in Burkina Faso. The current report explores this gap and assesses for the first time the level and distribution of detoxifying enzyme activities in *An*. *gambiae* mosquitoes from ten sentinel sites spread throughout most of the country and provides a detailed update on phenotypic resistance to the four classes of insecticides used in public health. Information on the occurrence and frequency of target-site resistance mutations (i.e. *kdr* and *Ace-1*) and species-specific composition within the *An. gambiae* complex is given for all sites, providing a comprehensive description of the extent and diversity of insecticide multiresistance phenotypes and mechanisms in major vector populations in Burkina Faso.

## Materials and Methods

### Study area

**Figure 1:**
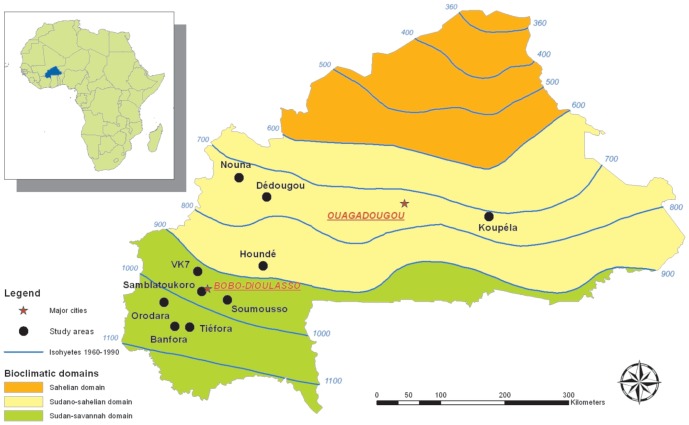
Map of Burkina Faso showing study sites.

The study was carried out in ten localities (Banfora, Dédougou, Houndé, Koupéla, Nouna, Orodara, Samblatoukoro, Soumousso, Tiéfora and Valley of Kou 7) belonging to two of the three ecological zones of Burkina Faso, West Africa: the Sudan savannah zone in the south and west of the country and the Sudano-sahelian domain, which extends throughout much of the central part of the country (Figure 1). No collection was conducted in the northernmost Sahelian domain where *An. arabiensis* predominates in adult collections [45]. In the Southwest, the rainy season extends from May to November with average yearly rainfall above 900 mm/year. In the central Sudano-sahelian area, the rainy season is shorter, extending from June to September with average yearly rainfalls between 600–900 mm mm/year. Climatic differences are reflected in different agricultural practices throughout the country, from arable to pastoral lands. However, in all sites, cotton cultivation is widespread, being the main market crop in Burkina Faso and consuming more than 90% of total pesticides used in the country [46].

### Mosquito collection and rearing

*An. gambiae* larvae were collected in August and September 2010 (rainy season) from natural breeding sites. In each locality, larval collections (all instars) were carried out in at least 10 different larval development sites and specimens were pooled per locality. Larvae were brought back to the insectaries at IRSS/Centre Muraz in Bobo-Dioulasso where they were reared to adults under standard controlled conditions (26±2°C, 80±10% RH and 12∶12 L-D). Larvae were fed with Tetramin™ baby fish food every day. Upon emergence, mosquitoes were morphologically identified using identification keys [47] and samples of 80 *An. gambiae* females were randomly picked from emerging mosquitoes from each locality and dry-frozen at −80°C for molecular and biochemical analysis. Remaining *An. gambiae* females were maintained on 5% sugar solution until they were used for insecticide susceptibility tests. The Kisumu strain of *An. gambiae sensu stricto*, a reference strains susceptible to all insecticides, was reared simultaneously under the same conditions and used as a control for insecticide bioassays.

### Insecticide susceptibility test

Adult susceptibility assays were carried out with five insecticides representative of all four classes of insecticides available for use in public health using insecticide-treated filter papers at the diagnostic dose as recommended by WHO [48], including two pyrethroids (type I: Permethrin 0.75% and type II: Deltamethrin 0.05%), one CM (Bendiocarb 0.1%), one OP (Fenithrotion 1%) and one organochlorine (DDT 4%). Bioassays were conducted with 2 to 5 days old, non blood-fed adult female mosquitoes. Batches of 20–25 test mosquitoes were exposed for 1 hour to insecticide-treated papers. Control mosquitoes (N = 20–25 females per test population and the Kisumu strain) were exposed for the same time to untreated filter papers. After exposure, mosquitoes were transferred into insecticide-free observation tubes and maintained on 5% sucrose solution at a temperature ranging from 25 to 28°C. Final mortality in test and control mosquitoes was recorded 24 h after exposure. The threshold of susceptibility was fixed at 98% mortality rate for the five active molecules according to the WHO's protocol [48]. Mortality rates below 80% were considered as indicative of insecticide resistance [48].

### Species identification and target site mutation genotyping

Thirty females *An. gambiae* were randomly picked from within the group of 80 specimens set apart from each test locality and they were used for molecular analyses. They were identified to species and molecular form using PCR as described by Fanello et al. [49], and this group was considered as representative of the mosquito populations being tested in each locality [15], [50]. Their genotype at the *kdr* locus was determined using the diagnostic tests described by Martinez-Torres et al. [51] and Ranson et al. [18]. PCR-RFLP was used to detect the presence of the G119S mutation in the *Ace-1* gene [26].

Genotypic frequencies at the *kdr* and *Ace-1* loci in *An. gambiae* populations were tested for goodness of fit to Hardy-Weinberg expectations using the Fischer exact tests implemented in GenePop (ver.3.4) software [52].

### Biochemical analysis

Detoxifying enzymes activities were measured on single mosquitoes (N = 50) from each test locality, which were stored at −80°C within 24 h from emergence (above). Each mosquito was ground on ice in 200 µl of distilled water and the homogenate was centrifuged at 1,4000 rpm for 2 mins. Two 10 µl replicates of supernatant were transferred into two adjacent wells of a microtitre plate for non-specific esterases (NSEs), gluthatione S-transferases (GSTs) and protein assays. Monooxygenases assays were performed with two 20 µl replicates of supernatant.

#### Proteins

Total protein content in 10 µl aliquots of mosquito homogenate was measured using the Bradford assay [53], in order to report detoxifying enzyme activity to protein value for each test mosquito. In each replicate well of the microtiter plate, 290 µl of Coomassie Plus Protein Assay Reagent solution [48] were added to 10 µl of centrifuged mosquito homogenate and the mixture was incubated at room temperature for 5 min. The endpoint absorbance was read at 590 nm. Protein values were calculated using a standard curve of absorbance of bovine serum albumin and served as a correction factor for the enzyme analysis.

#### Non-Specific Esterases (NSEs)

Non-specific esterase activity was measured using α-naphtol acetate (αNa) and β-naphtol acetate (βNa) [48]. In each replicate well, 90 µl of phosphate buffer (PBS, pH = 6.5) and 100 µl of 0.6 M αNa (or βNa) were added to 10 µl of centrifuged mosquito homogenate. After 30 min incubation, 100 µl of Fast Garnett BC solution (8 g Fast Garnett Salt + 10 ml distilled water) was added to stop the reaction. The concentration of the final product was determined at 550 nm as an endpoint calculated from standard curves of α- and β-Naphtol, respectively.

#### Glutathione-S-Transferases (GST)

To measure glutathione-S-transferase (GST) activity in mosquitoes, 200 µl of GSH/CDNB working solution (100 µl of an extemporaneous solution of 0.6% weight/volume reduced glutathione in 0.1 M sodium phosphate buffer pH = 6.5+0.013 g of 1-chloro-2, 4-dinitrobenzene diluted in 1 ml of 70% methanol) were added to each replicate of mosquito homogenate [48]. The reaction was read at 340 nm immediately as a kinetic assay for 5 minutes. An extinction coefficient of 5.76 mM^−1^ (corrected for a path length of 0.6 cm) was used to convert absorbance values to moles of product. GST specific activity was reported as the rate of formation of GSH produced in mmol.min^−1^.mg^−1^ protein.

#### Oxydases (Cytochrome P450)

Cytochrome P450 activity was determined using the heme-peroxidase assay according to Brogdon et al. [54]. The assay detects the elevation in the amount of heme, which is then converted into equivalent units of cytochrome P450. Eighty µl of 0.625 M potassium phosphate buffer (pH = 7.2) were added to 20 µl of mosquito homogenate together with 200 µl Tetramethyl Benzidine solution (0.011 g 3,3′,5,5′ Tetramethyl Benzidine in 5 ml of 70% methanol +15 ml sodium acetate buffer 0.25 M pH = 5.0); 25 µl of 3% hydrogen peroxide were then added and the mixture was incubated for 30 min at room temperature. Absorbance was read at 630 nm and values calculated from a standard curve of cytochrome C.

#### Data analysis

Mean absorbance values of replicate wells for each tested mosquito were converted into enzyme activity and divided by the protein values. The median enzymatic activity was calculated for each test mosquito population and the distribution of enzyme activities was compared between the Kisumu reference strain and the field populations using non-parametric Mann-Whitney tests.

## Results

### Resistance status

**Figure 2:**
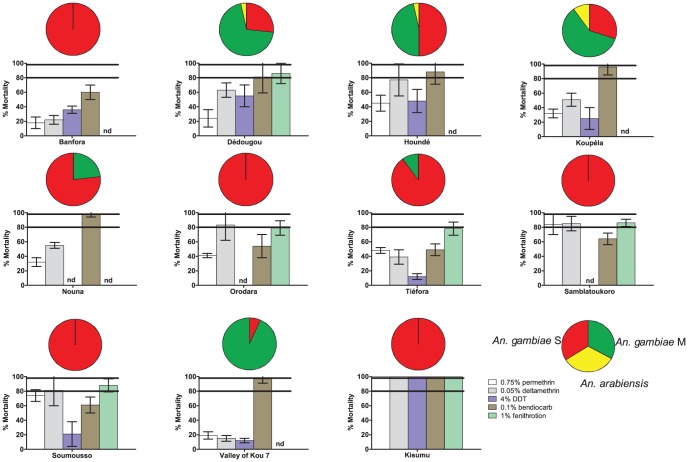
Insecticide mortality rates and species composition in ten *Anopheles gambiae* s.l. test populations from Burkina Faso collected in the 2010 rainy season. For each locality, pie charts (above) indicate the relative frequency of each taxon within *An. gambiae* s.l. (N = 30 specimens per locality, see text) and bar charts (below) show percentage mortality 24 hours after a 1-hour exposure to the diagnostic doses of insecticide (N = 100 per population). Mortality thresholds (98% and 80%, as recommended by WHO [48]) are shown on each bar chart (horizontal black lines). nd = not determined.

Mortality rate in unexposed controls from each site and the Kisumu laboratory strain was less than 5% in all cases and no correction of test sample data was required. In some sites, insufficient sample sizes precluded the realization of the whole set of bioassays. However, in all sites, mosquitoes demonstrated multiple resistance phenotypes, showing reduced mortality rates to several insecticidal compounds at the same time, although with considerable site-to-site variation (Figure 2). Resistance to DDT (tested at 4%), permethrin (0.75%) and deltamethrin (0.05%) was most widespread, and frequently observed together within the same sample. Mortality rates to all three insecticides were indeed below 50% in at least 4 out of the ten sites investigated (i.e., Banfora, Koupela, Tiefora and VK7), reflecting high levels of resistance. However, in other sites (e.g., Hounde, Orodara, Samblatoukoro and Soumousso), resistance to deltamethrin, and to some extent to permethrin as well, was less marked with mortality rates above 80% being observed, suggesting reduced susceptibility which deserves further monitoring in order to establish the phenotypic status of resistance in these vector populations. Resistance to CMs (i.e., bendiocarb 0.1%) and OPs (i.e., fenithrotion 1%) was less frequent in our samples. However, mortality rates below the 80% threshold were recorded for bendiocarb in all localities within the cotton-intensive cultivation belt in the south-western part of our study area (i.e., Banfora, Orodara, Tiefora, Samblatoukoro and Soumousso) except in the rice-fields area of VK7 where the vector population seems still completely susceptible to this insecticide (mortality = 100%, Figure 2). Hence, resistance to bendiocarb seemed to assort independently of pyrethroids and DDT resistance. Finally, everywhere where it has been tested (i.e., 5 sites, Figure 2), there was evidence for reduced mortality following exposure to the OP fenitrothion, although none of the vector population tested appeared as resistant to this insecticide (i.e. mortality rates above 80%). Here again, future studies should insure close monitoring of the natural vectors populations susceptibility to this compound.

### Species and molecular forms of *Anopheles gambiae*

Mosquitoes were analyzed by PCR for identification of sibling species within the *An. gambiae* complex and of the M and S molecular forms within *An. gambiae* s.s.. Mosquito populations were composed of a mixture of *An*. *gambiae* s.s. and *An*. *arabiensis* in most of the sampled localities (Figure 2). *An. arabiensis* was found at low frequency (<10%) in the northernmost localities (i.e., Dédougou, Houndé and Koupéla) where it occurred together with the M and S forms of *An. gambiae* s.s.. The M form of *An. gambiae* s.s. was mainly found in the Soudano-Sahelian zone (Dédougou, Houndé, Koupéla and Nouna) and was highly predominant in the irrigated rice fields area around VK7 where it accounted for 93% of the sampled specimens. The S form of *An. gambiae* s.s. was found in all localities; it was predominant in 7 out of 10 sampled localities and was the only one member of the complex detected in four out of six localities within the Soudan savannah area (Banfora, Orodara, Samblatoukoro and Soumousso). These distributions are in agreement with previous reports [44], [55].

### Detection of resistance mutations by PCR

Allelic and genotypic frequencies at the *kdr* and *Ace-1* loci are shown in Table 1 and Table 2, respectively. The *kdr* 1014S allele was not found in our samples. However, the *kdr* 1014F resistant allele was found in both molecular forms of *An. gambiae* s.s. and in *An. arabiensis*, floating at various allelic frequencies. The frequency of the resistant *kdr* allele was highest in the S form of *An*. *gambiae* s.s., occurring at a frequency generally above 0.5 and almost fixed in some localities (e.g., Orodara and Tiefora, with frequencies above 95%, Table 1). In most cases, significant departure from Hardy-Weinberg proportions was observed. In the M form, the resistant *kdr* allele was also widespread, being detected in all sampled populations, although limited sample sizes precluded precise estimates of its frequency in our samples. The *kdr* 1014F allele was also found in 2 out of the 5 specimens of *An. arabiensis* that were included in our study, suggesting it is widespread in this species as well.

**Table 1:** Allelic and genotypic frequencies at the *kdr* locus in *An. gambiae* s.l. populations from sites in Burkina Faso (2010).

			Genotypes					
			1014L	1014L	1014F			
	Sites	N	1014L	1014F	1014F	f(1014F)	[95%CI]	p(HW)
*An. gambiae*	Banfora	30	4	1	25	0.85	[0.79–0.91]	<0.0001
S form	Dédougou	8	4	0	4	0.50	[0.33–0.67]	0.0054
	Houndé	15	2	1	12	0.83	[0.74–0.92]	0.0192
	Koupéla	9	5	0	4	0.44	[0.28–0.60]	0.0029
	Nouna	7	2	1	4	0.64	[0.46–0.82]	0.4406
	Orodara	30	0	2	28	0.97	[0.94–1.00]	1
	Samblatoukoro	27	3	0	24	0.89	[0.84–0.94]	<0.0001
	Soumousso	30	7	0	23	0.77	[0.70–0.84]	<0.0001
	Tiéfora	30	1	1	28	0.95	[0.92–0.98]	0.0508
	VK7	2	0	2	0	0.50	[0.15–0.85]	1
*An. gambiae*	Banfora	0	-	-	-	-	-	-
M form	Dédougou	21	8	2	11	0.57	[0.47–0.67]	0.0002
	Houndé	14	11	2	1	0.14	[0.05–0.23]	0.2178
	Koupéla	18	13	0	5	0.28	[0.18–0.38]	<0.0001
	Nouna	23	10	3	10	0.50	[0.40–0.60]	0.0005
	Orodara	0	-	-	-	-	-	-
	Samblatoukoro	3	0	1	2	0.83	[0.62–1.00]	-
	Soumousso	0	-	-	-	-	-	-
	Tiéfora	0	-	-	-	-	-	-
	VK7	28	3	15	10	0.63	[0.54–0.72]	0.6891
*An. arabiensis*	Banfora	0	-	-	-	-	-	-
	Dédougou	1	1	0	0	0	-	-
	Houndé	1	0	1	0	0.50	[0.00–1.00]	-
	Koupéla	3	2	0	1	0.33	[0.06–0.60]	0.2000
	Nouna	0	-	-	-	-	-	-
	Orodara	0	-	-	-	-	-	-
	Samblatoukoro	0	-	-	-	-	-	-
	Soumousso	0	-	-	-	-	-	-
	Tiéfora	0	-	-	-	-	-	-
	VK7	0	-	-	-	-	-	-

Note that the *kdr* 1014S resistant allele was not detected in our samples and is therefore not reported in the Table.

N: number of mosquitoes;

f(1014F): frequency of the 1014F resistant *kdr* allele;

p(HW): probability of the exact test for goodness of fit to Hardy-Weinberg equilibrium;

[95%CI]: 95% confidence interval;

‘-’: not determined.

**Table 2:** Allelic and genotypic frequencies at the *ace-*1 locus in *An. gambiae* s.l. populations from sites in Burkina Faso (2010).

			Genotypes					
			119G	119G	119S			
	Sites	N	119G	119S	119S	f(119S)	[95%CI]	p(HW)
*An. gambiae*	Banfora	30	18	12	0	0.20	[0.13–0.27]	0.560
S form	Dédougou	8	5	3	0	0.21	[0.07–0.35]	1
	Houndé	15	10	5	0	0.17	[0.08–0.26]	1
	Koupéla	9	9	0	0	0	-	-
	Nouna	7	5	2	0	0.14	[0.01–0.27]	1
	Orodara	30	14	16	0	0.27	[0.19–0.35]	0.075
	Samblatoukoro	27	14	13	0	0.24	[0.16–0.32]	0.283
	Soumousso	30	21	9	0	0.15	[0.09–0.21]	1
	Tiéfora	30	19	11	0	0.18	[0.11–0.25]	0.551
	VK7	2	1	1	0	0.25	[0.00–0.55]	-
*An. gambiae*	Banfora	0	-	-	-	-	-	-
M form	Dédougou	21	20	1	0	0.02	[0.00–0.05]	-
	Houndé	14	14	0	0	0	-	-
	Koupéla	18	18	0	0	0	-	-
	Nouna	23	23	0	0	0	-	-
	Orodara	0	-	-	-	-	-	-
	Samblatoukoro	3	3	0	0	0	-	-
	Soumousso	0	-	-	-	-	-	-
	Tiéfora	0	-	-	-	-	-	-
	VK7	28	28	0	0	0	-	-
*An. arabiensis*	Banfora	0	-	-	-	-	-	-
	Dédougou	1	1	0	0	0	-	-
	Houndé	1	1	0	0	0	-	-
	Koupéla	3	3	0	0	0	-	-
	Nouna	0	-	-	-	-	-	-
	Orodara	0	-	-	-	-	-	-
	Samblatoukoro	0	-	-	-	-	-	-
	Soumousso	0	-	-	-	-	-	-
	Tiéfora	0	-	-	-	-	-	-
	VK7	0	-	-	-	-	-	-

N: number of mosquitoes;

f(119S): frequency of the 119S resistant *ace-1* allele;

p(HW): probability of the exact test for goodness of fit to Hardy-Weinberg equilibrium;

[95%CI]: 95% confidence interval;

‘-’: not determined.

The *Ace-1* ^R^ 119S allele was detected in both the M and S forms of *An. gambiae s.s.*, and was not observed in the few (N = 5) *An. arabiensis* analysed. Again, the resistant allele was much more common in the S form of *An. gambiae*, where it was found floating at a frequency generally below 0.25, than in the M form, where only one occurrence of the allele was found (Table 2). In all cases, Hardy-Weinberg proportions were respected although the resistant 119S allele was never observed in a homozygous state in our populations.

An important result is the concomitant detection of the *kdr* 1014F and *Ace-1* ^R^ 119S alleles in 47 *An. gambiae* S form.

### Biochemical assays

#### Non-specific esterases (NSE)

**Figure 3:**
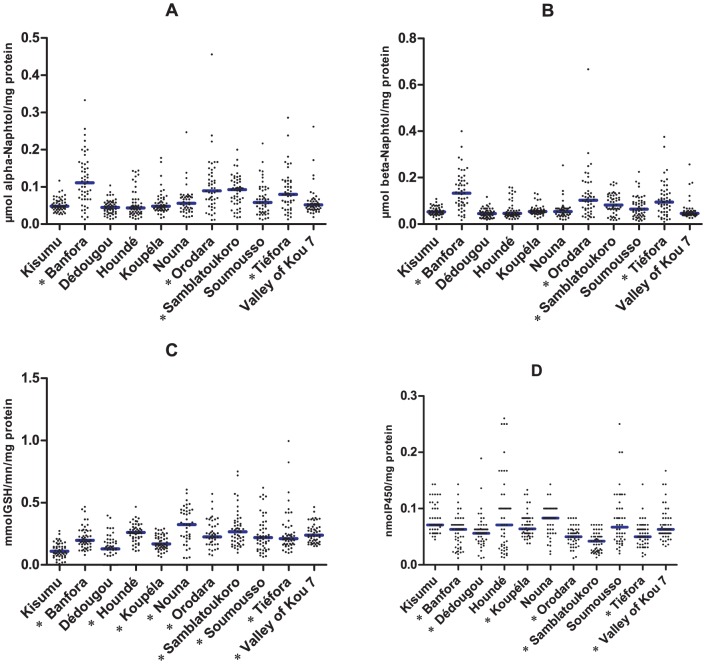
Detoxifying enzyme activities in *An. gambiae* s.l. mosquitoes collected form ten localities in Burkina Faso (2010). A) Non-Specific α Esterase activity, B) Non-Specific β Esterase activity, C) Glutathione-S-Transferase (GST) activity, D) P450 activity. Stars denote significant difference in activity level when compared to the *An. gambiae* Kisumu reference strain.

Figures 3A and 3B show the results of NSE activity with α and β naphtyl acetate as a substrate, respectively. The activity in the Kisumu susceptible strain ranged from 0.02 to >0.11 µmol α-naphthol produced/mg protein (median = 0.048±0.018). The same range of activities was observed with βNa (median = 0.050±0.019 µmol β-naphthol produced/mg protein). Higher levels of esterase activity (using either α or β naphtyl acetate as a substrate) were detected in the mosquito populations collected in Banfora, Orodara, Tiéfora and Samblatoukoro compared with the Kisumu strain (Mann-Whitney test, p<0.0001 in all cases), with a greater proportion of individuals showing an increased NSE activity level. Although it was not possible to identify single mosquitoes to species and molecular form due to logistical constraints, the highest levels of esterase activity were observed in localities where the S form was predominant.

#### Gluthathione-S-Transferases (GST)

The distribution of enzymatic activities for GST in the laboratory and field populations is shown in Figure 3C. The activity in the Kisumu strain ranged from 0.022 to 0.273 mmol GSH/min/mg protein (median = 0.112±0.057). A significantly higher level of GST activity was observed in all field populations when compared to the Kisumu strain (Mann-Whitney test, p<0.001 in all cases), except in Dédougou (median = 0.146±0.092).

### Oxydases (Cytochrome P450)

The distribution of oxydase (P450) activity in the laboratory and field samples is shown in Figure 3D. The activity in the Kisumu strain ranged from 0.045 to 0.143 nmol P450/mg protein (median = 0.071±0.027). The highest level of P450 activity were detected in field populations at Houndé and Nouna but were not statistically different from the Kisumu susceptible strain (Mann-Whitney test, P>0.05 in all cases). However, in Banfora, Dédougou, Koupéla, Orodara, Tiéfora, Samblatoukoro and VK7 the median P450 activity was significantly lower than that of the Kisumu strain (Mann-Whitney test, P<0.03 in all cases). A significantly higher level of P450 activity was observed in all field populations when compared to the Samblatoukoro sample (Mann-Whitney test, p<0.001 in all cases).

## Discussion

In Burkina Faso, country-wide surveys associating insecticide susceptibility tests and molecular assays for the detection of mutations associated with insecticide resistance have been ongoing since 1999 [11], [30], [44], [56]. All these studies documented increasing levels of insecticide resistance in field populations of the M and S forms of *An. gambiae* s.s. and *An. arabiensis* throughout the country, together with a dramatic rise in frequency of the *kdr* 1014F allele over the last decade and presence of the resistant *Ace-1* ^R^ 119S allele in both molecular forms [57]. In our study, we provided further evidence that insecticide multi-resistance is a common phenotype within *An. gambiae* populations from Burkina Faso, and that target-site mutations are widespread. In the S form of *An. gambiae*, the resistant *kdr* 1014F and *Ace-1* ^R^ 119S alleles were found within the same specimen, further advocating for the combination of different resistance mechanisms at the individual level. Resistance to DDT and permethrin was widespread, as formerly reported. However, reduced susceptibility to deltamethrin was observed in all sampling localities whereas this was not the case in previous reports documenting susceptibility to insecticides in mosquito populations collected before 2006 [9]. This new result underlines the rapid dynamics of evolution of resistance phenotypes which is ongoing in this area of West-Africa where cotton cultivation is widespread [11], [46]. The incrimination of agricultural use of pesticides in the selection for insecticide resistance in malaria vector mosquitoes has been documented in several countries in West Africa, including Burkina Faso [9], [11], [41], [56], [58]. Bendiocarb (CM) could provide a suitable alternative to the use of pyrethroids for resistant malaria vector control [59]. Unfortunately however, resistance to bendiocarb (used at the diagnostic dose of 0.1%) was observed in wild *An*. *gambiae* populations from five out of the ten sites we investigated in this study, with only three localities where *An. gambiae* populations remained fully susceptible to this compound. It is therefore highly likely that the use of bendiocarb as an alternative to pyrethroids is jeopardized in Burkina Faso. Interestingly, resistance to bendiocarb was higher in localities where the S form was predominant, suggesting that CM resistance might be more common in the S than in the M form, for genetic and/or ecological reasons. Presence of the *Ace-1* ^R^ 119S allele in both molecular forms of *An. gambiae* further strengthens this view. It is noteworthy that in the S form, where the 119S allele is widespread, it is rarely found at the homozygous state. This is consistent with the occurrence in these natural vector populations of a duplication of the *Ace-1* gene, resulting in the *Ace-1^D^* allele creating ‘fixed heterozygotes’ fingerprints [60], [61]. It has been demonstrated in *Culex pipiens* that *Ace-1* duplication reduces the high fitness cost associated to the G119S mutation [60], [62] and might therefore foster the spread of the resistant allele in natural vector populations [57], [63].

An important result presented above is the first data documenting variation in detoxifying enzyme activities within and between natural vector populations in the country, suggesting metabolic resistance mechanisms might contribute to the overall pattern of insecticide resistance. As mentioned above, we were unable to identify the specimens used for these assays to the species/form level, and it is therefore possible that, in areas where different species or forms are sympatric such as in Houndé, using a mixture of the M and S forms of *An. gambiae* and *An. arabiensis* could affect the overall level of enzymatic activities we have measured to an unknown extent. Nevertheless, some robust trends could be drawn out of our results. High levels of GST activities were widespread. The primary function of GSTs is generally considered to be the detoxification of endogenous and xenobiotic compounds or their secondary metabolites, including insecticides. GSTs were indeed shown to catalyse OP and DDT metabolism directly, while they might play a minor role in pyrethroid resistance through the detoxification of lipid peroxidation products induced by pyrethroids [16], [64]. GSTs might therefore complement the phenotypic effect of *kdr* mutations towards increasing resistance levels to pyrethroids and DDT and broadening the resistance spectrum to unrelated compounds [64]. Elevated levels of NSE activity were also detected in some sites: increased NSE activity was observed in Banfora, Orodara, Samblatoukoro, Tiéfora and, to a lower extend Soumousso mosquito populations which consisted mainly of the S form of *An. gambiae*. In contrast, in those sites where the M form of *An. gambiae* is predominant, NSE activity was not significantly different from the Kisumu reference strain. Enhanced NSE activity due to the duplication of insecticide-sequestering esterase genes has been demonstrated to be a mechanism providing resistance to OP and CM in a number of arthropod species including mosquitoes, ticks, aphids and cockroaches [16]. Hence, NSEs could further contribute to insecticide resistance, especially in the S form of *An. gambiae* from Burkina Faso, where elevated NSE activity was observed together with high frequencies of target-site resistance mutations. Recent microarray studies have shown that esterases play no role or very little in pyrethroid and DDT resistance in field populations of *An. gambiae* [41], [43], [65]. Hence, elevated NSE might also be caused by other ecological or genetic differences between the field strains and the Kisumu laboratory strain, not because of the resistance phenotype in these populations. There was however no evidence for an overall increase in cytochrome P450 activity in our samples, as compared to the Kisumu reference strain assessed simultaneously. However, biochemical assays only provide an overall estimate of P450 enzymatic activity and it is possible that different genes within this multigenic family are differentially expressed in different mosquito populations, as microarray studies conducted in West and Central African populations of *An. gambiae* revealed [41], [43]. Bioassays carried out with the synergist PBO, an inhibitor of P450s, should further allow incrimination of P450s in the resistance profile we observed [66].

The presence of multiple resistance mechanism in *An. gambiae* from Burkina Faso may constitute an obstacle for the future success of malaria control programmes based on LLINs or IRS although the operational impact of the concomitant presence of multiple resistance mechanisms on the efficacy of insecticide-based vector control still needs to be demonstrated. However, by documenting the extent and diversity of insecticide resistance phenotypes and the putative combination of their underlying mechanisms in *An. gambiae* mosquitoes, our work prompts for new alternative strategies to be urgently developed for the control of major malaria vectors in Burkina Faso and more globally in West Africa.
